# Sirtuins in kidney diseases: potential mechanism and therapeutic targets

**DOI:** 10.1186/s12964-023-01442-4

**Published:** 2024-02-12

**Authors:** Qi Jin, Fang Ma, Tongtong Liu, Liping Yang, Huimin Mao, Yuyang Wang, Liang Peng, Ping Li, Yongli Zhan

**Affiliations:** 1 Guang’anmen Hospital, China Academy of Chinese Medical Sciences, Beijing, China; 2 China-Japan Friendship Hospital, Institute of Clinical Medical Sciences, Beijing, China

**Keywords:** Sirtuins, Kidney diseases, Podocyte, Renal tubular epithelial cells, Endothelial cells, Macrophages

## Abstract

Sirtuins, which are NAD^+^-dependent class III histone deacetylases, are involved in various biological processes, including DNA damage repair, immune inflammation, oxidative stress, mitochondrial homeostasis, autophagy, and apoptosis. Sirtuins are essential regulators of cellular function and organismal health. Increasing evidence suggests that the development of age-related diseases, including kidney diseases, is associated with aberrant expression of sirtuins, and that regulation of sirtuins expression and activity can effectively improve kidney function and delay the progression of kidney disease. In this review, we summarise current studies highlighting the role of sirtuins in renal diseases. First, we discuss sirtuin family members and their main mechanisms of action. We then outline the possible roles of sirtuins in various cell types in kidney diseases. Finally, we summarise the compounds that activate or inhibit sirtuin activity and that consequently ameliorate renal diseases. In conclusion, targeted modulation of sirtuins is a potential therapeutic strategy for kidney diseases.

Video Abstract

**Supplementary Information:**

The online version contains supplementary material available at 10.1186/s12964-023-01442-4.

## Introduction

Sirtuins comprise a family of nicotine adenine dinucleotide (NAD^+^)-dependent class III histone deacetylases that are closely associated with organismal health and disease progression [1]. Sirtuins can be traced back 40 years to the silent information regulator 2 (Sir2) in *Saccharomyces cerevisiae*. Sir2 represses transcription at ribosomal DNA sites and telomeres, extending yeast lifespan by improving genomic instability, and further studies have revealed that Sir2 has NAD^+^-dependent histone deacetylase activity. As homologous genes of Sir2 have been gradually isolated from animals, plants, and bacteria. Sir2 homologous proteins in all species are collectively referred to as sirtuins [2]. To data, seven sirtuin family members have been identified in mammals, namely, Sirt1-Sirt7. The structures of these members included identical central structural regions. However, differences in their respective active sites result in specific biological functions [3]. Initially, sirtuins were defined as histone deacetylases, but with further studies, sirtuins have been shown to have multiple enzymatic activities, including mono-ADP-ribosyltransferase, deacylase, decrotonylase, demalonylase, and desuccinylase activities. Sirtuins deacetylate non-histone proteins and regulate cellular processes [4]. Sirtuins depend on NAD^+^ for their activity, which is transformed into nicotinamide (NAM) in the presence of sirtuins. NAM is then transformed into nicotinamide mononucleotide (NMN) by the action of intracellular nicotinamide phosphoribosyltransferase (iNAMPT), which in turn is catalysed into NAD by the critical rate-limiting enzyme nicotinamide mononucleotide adenylyltransferase (NMNAT), and the cycle repeats [5]. The level of NAD^+^ is closely associated with disease progression. Furthermore, several studies have confirmed that the level of NAD^+^ decreases with the progression of renal disease and that enhancement of NAD^+^ has an ameliorating effect [6].

The sirtuin family has received much attention in the past 20 years, attributed to their involvement in the regulation of various critical biological processes in preclinical and clinical models, including oxidative stress, inflammation, mitochondrial homeostasis, autophagy, DNA damage repair, and procedures and functions that are essential for maintaining cellular and organismal homeostasis [7]. Activation of sirtuins can delay the progression of several renal diseases, including diabetic kidney disease (DKD), acute kidney injury (AKI), and hypertensive nephropathy. In DKD mice, Sirt1 promotes the activity of forkhead box O (FOXO) 3a, exerts antioxidant effects, and reduces oxidative stress injury in DKD mice [8]. Overexpression of Sirt7 also reduces inflammation and improves renal function in DKD [9]. Lack of Sirt3 further exacerbates the pathological damage of AKI, while overexpression of Sirt3 promotes optic atrophy 1 (OPA1)-mediated mitochondrial fusion and alleviates mitochondrial damage in AKI [10]. Sirt1 regulates autophagy and delays the progression of AKI by deacetylating of the autophagy regulator Beclin1 [11]. In hypertensive nephropathy, an increase in the number of DNA double-strand breaks (DSBs) is accompanied by a decrease in Sirt6 expression [12]. Although many studies have confirmed the modulatory role of sirtuins in renal disease, their exact role remains unclear.

Sirtuins have long been considered therapeutic targets for various diseases, and small molecules or natural compounds that regulate sirtuins are promising potential therapeutic agents [13]. Sirtuins target and regulate various biological processes in kidney cells and are involved in the progression of various renal diseases. Podocyte-specific knockdown of Sirt6 exacerbates podocyte injury and proteinuria in adriamycin-induced nephropathy and DKD. In addition, Sirt6 overexpression protects against podocyte apoptosis and inflammatory injury by deacetylating H3K9, inhibiting Notch1 and Notch4 transcription, and enhancing autophagy [14]. Mice with specific knockdown of Sirt3 in proximal renal tubular epithelial cells (RTECs) were more likely to exhibit increased acetylation of mitochondrial proteins and enhanced renal fibrosis than normal mice. In contrast, activation of Sirt3 improved their acetylation levels and delayed renal fibrosis [15]. Continuous research on sirtuin family members has led to the development of modulators targeting sirtuins, such as resveratrol and curcumin, which improve renal disease by activating Sirt1 and Sirt3. In contrast, synthetic sirtuin inhibitors, such as AK-1, effectively alleviate renal disease by inhibiting Sirt2 progression [16]. In this review, we summarise the studies on the sirtuin family regulation of renal cells, and thus, the improvement of renal diseases, by describing the various functions of sirtuin family members and highlighting the therapeutic potential of sirtuin modulators in renal diseases.

## The origin and function of the sirtuin family

**Fig. 1:**
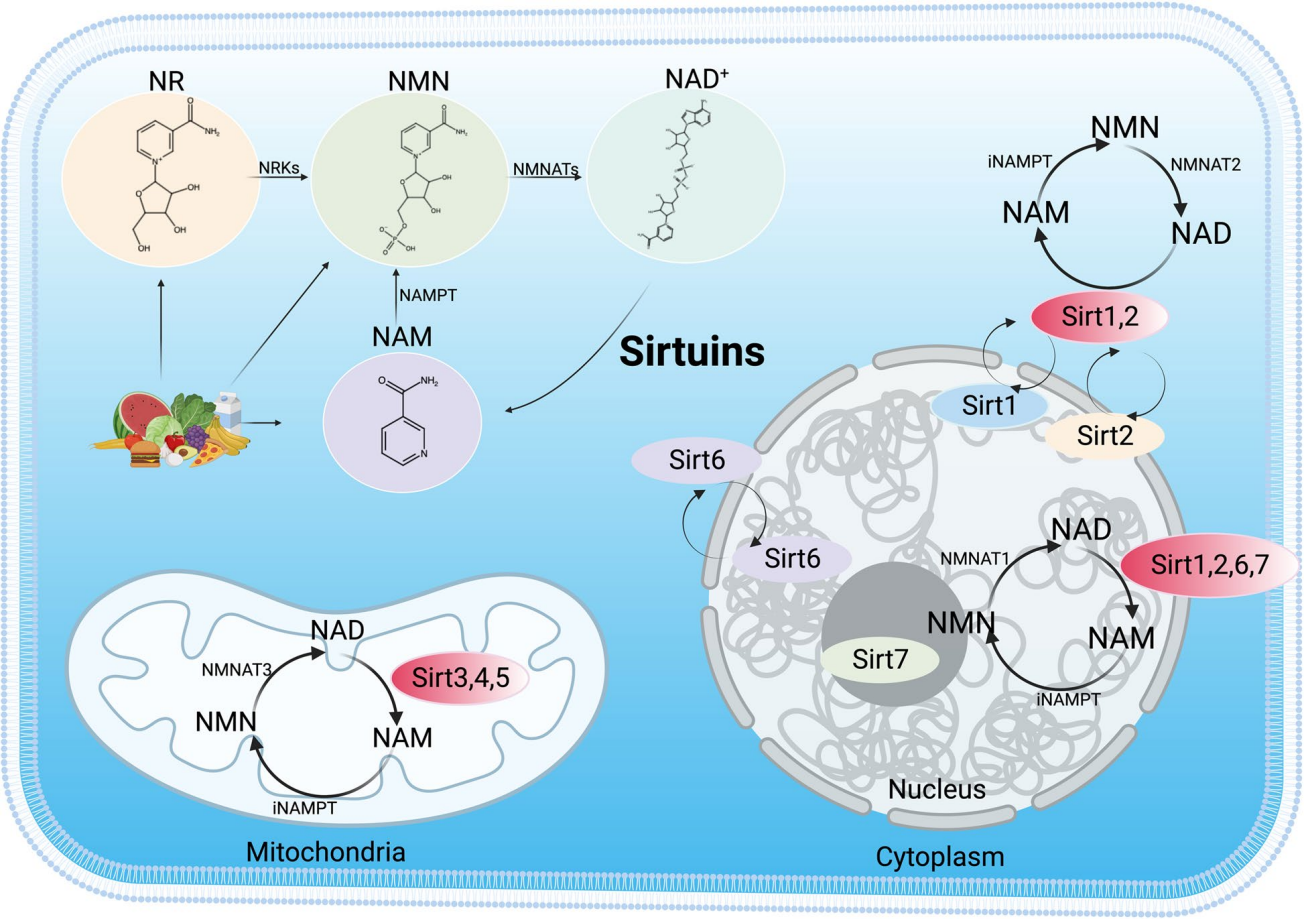
Location and distribution of sirtuins. NR, nicotinamide riboside; NMN, nicotinamide mononucleotide; NAD^+,^ nicotine adenine dinucleotide; NAM, nicotinamide; iNAMPT, intracellular nicotinamide phosphoribosyltransferase; Nmnat, nicotinamide mononucleotide adenylyltransferase. (Created with BioRender.com).

**Fig. 2:**
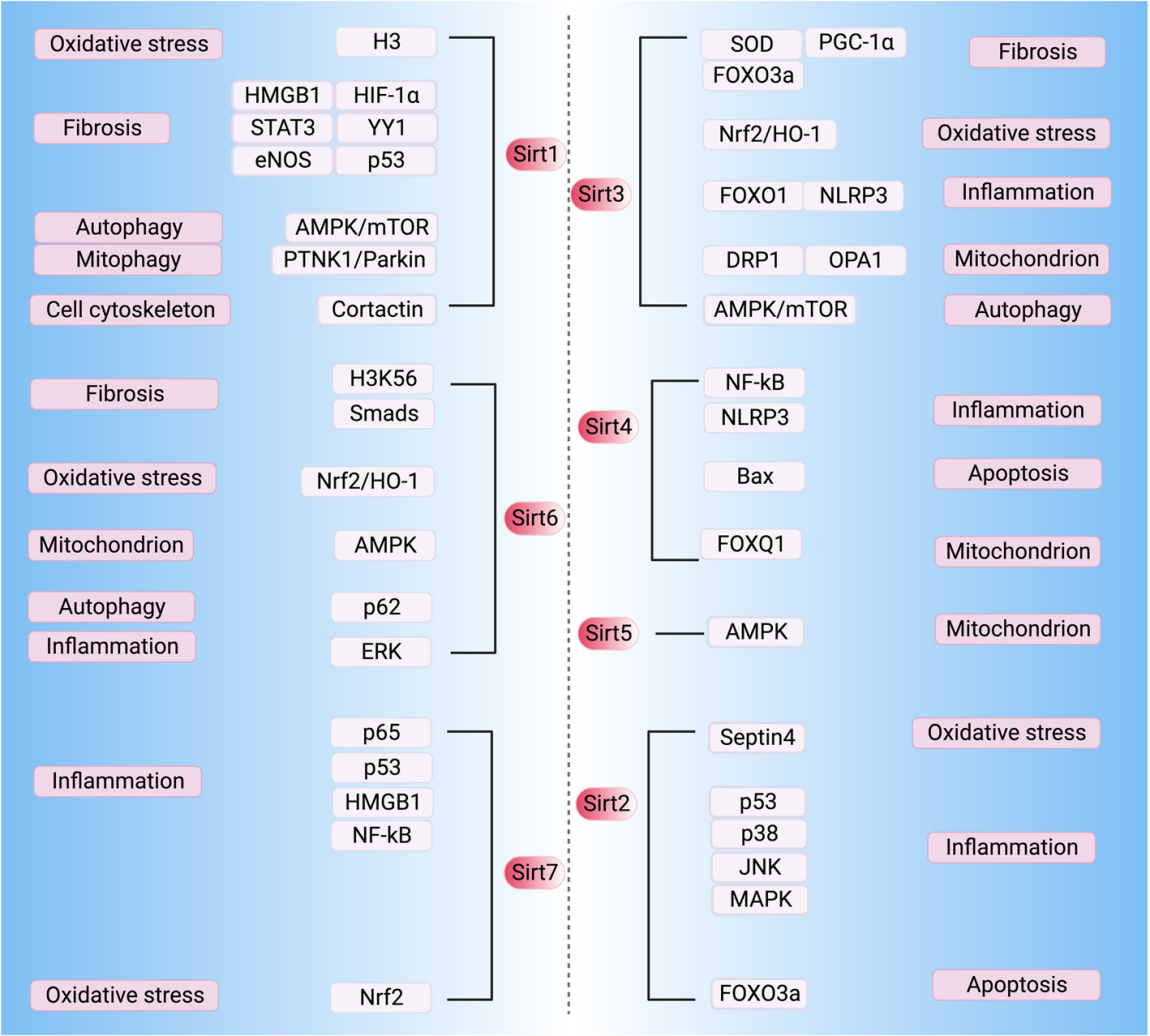
Primary targets and cellular processes regulated by sirtuins in kidney diseases. HMGB1, high-mobility group box 1; HIF-1ɑ, hypoxia-inducible factor-1; STAT3, signal transducer and activator of transcription 3; YY1, Yin yang 1; eNOS, endothelial nitric oxide synthase; AMPK, AMP-activated protein kinase; mTOR, mammalian target of rapamycin; PINK1, PTEN-induced kinase 1; H3K56, histones3 lysine56; Nrf2, nuclear factor-erythroid 2-related factor 2; HO-1, heme oxygenase-1; ERK, extracellular signal-regulated kinase; NF-κB, nuclear factor kappa B; SOD, superoxide dismutase; PGC-1ɑ, peroxisome proliferator-activated receptor-gamma coactivator 1-alpha; NLRP3, NOD-like Receptor Pyrin Domain Containing 3; DRP1, dynamin-related protein 1; OPA1, optic atrophy 1; JNK, c-Jun N-terminal kinase; MAPK, mitogen-activated protein kinase. (Created with BioRender.com)

Sirtuins are a family of highly evolutionarily conserved NAD^+^-dependent class III histone deacetylase signalling proteins that are widely found in prokaryotes and eukaryotes. The sirtuin family comprises seven homologous members: Sirt1-Sirt7. They are distributed across different parts of the cell. Sirt1 and Sirt2 are in the nucleus and cytoplasm, Sirt6 is in the nucleus, Sirt7 is in the nucleolus, and Sirt3, 4, and 5 are located in the mitochondria [17]. The sirtuin structure consists of a central catalytic region, an N-terminal region, and a C-terminal region. Although subtle differences in the binding sites may exist, the catalytic core region of the sirtuin family is structurally conserved. Notably, the N- and C-termini of sirtuins differ considerably in length, chemical composition, and sensitivity to post-translational modifications compared with the conserved catalytic core region [18]. Sirtuins have different biological functions because of their different binding sites and subcellular localisations. For example, Sirt1, Sirt2, Sirt3, Sirt5, Sirt6, and Sirt7 all have NAD^+^-dependent deacetylase activity that mediates the deacetylation of histones and non-histones. Sirt4 [19] and Sirt6 have mono-ADP-ribosyltransferase activity [20], and Sirt5 is also a desuccinylase [21]. Sirtuins require NAD^+^ as a catalytic cofactor and can hence be inhibited by NADH; therefore, sirtuins are particularly sensitive to the intracellular NAD^+^/NADH ratios [22]. Sirtuins are involved in a variety of metabolic regulation and biological processes, such as cell survival, apoptosis, proliferation, cellular senescence, stress response, inflammation, oxidative stress, mitochondrial production, genome stabilisation and metabolism. The complexity of the interactions between sirtuins provides a degree of support for their role as essential regulators of cellular biology [23] (Figs. 1 and 2).

### Sirtuins in the nucleus

#### Sirt1

Sirt1 is mainly localised in the nucleus; however, in response to certain stimuli, Sirt1 translocates from the nucleus to the cytoplasm [24]. It is involved in the regulating of a variety of biological processes, including oxidative stress [25], inflammation [26], mitochondrial metabolic disorders [27], autophagy [11], DNA damage repair [28], and telomere maintenance [29]. Compared with normal mice, mitochondrial dysfunction and lethality are significantly higher in systemic Sirt1 knockout mice after AKI [30]. It deacetylates histones and non-histones and maintains normal cellular function. Sirt1 regulates acetyl-histone H3 expression in a high glucose (HG) environment and attenuates streptozotocin (STZ)-induced renal oxidative damage in diabetic mice [31]. Cytoplasmic cortactin is important for maintaining the actin cytoskeleton. Sirt1 protects podocytes and repairs glomerular damage by activating cortactin deacetylation in the nucleus, which drives the localisation of acetylated cortactin to the cytoplasm and maintains actin cytoskeleton integrity [32]. Sirt1 also deacetylates the transcription factor Yin Yang 1 (YY1) to improve HG-induced epithelial-mesenchymal transition (EMT) [33]. Acetylation of high-mobility group box 1 (HMGB1) protein is a crucial process prior to its transfer from the nucleus to the cytoplasm and extracellular secretion in renal cells, which accelerates the progression of renal disease. Sirt1 deacetylates the HMGB1 lysine site and inhibits downstream inflammatory transmission [34]. Ferroptosis is an iron-dependent process of lipid peroxidation, and p53 is involved in the regulation of ferroptosis. In a renal fibrosis model, p53 expression and acetylation levels increased, whereas Sirt1 inhibited the progression of ferroptosis by inducing deacetylation of p53 [35]. Compared with young mice (5 weeks old), aged mice (24 months old) exhibited reduced Sirt1 expression. They exhibited higher deposition of extracellular matrix (ECM), and overexpression of Sirt1, through deacetylation of hypoxia-inducible factor-1 (HIF-1α), effectively alleviated hypoxia-induced ROS production, mitochondrial damage, and ECM protein production, with a protective effect on the tubulointerstitium of aged kidneys [36].

In addition to deacetylation, Sirt1 is involved in additional modifications, such as phosphorylation, ubiquitination, and other critical physiological and pathological processes. Sirt1 induces p65 nuclear factor kappa B (NF-κB) and signal transducer and activator of transcription (STAT)-3 dephosphorylation and deacetylation, reducing the inflammatory response, oxidative stress, and EMT in DKD [37]. In a unilateral ureteral obstruction (UUO) mouse model, activation of Sirt1 signaling was accompanied by an increase in phosphorylated endothelial nitric oxide synthase (eNOS) levels, and Sirt1 interacted with eNOS to improve the UUO model for scoring renal fibrosis [38]. Oxidative stress in DKD leads to Sirt1 ubiquitination, which promotes Sirt1 degradation, whereas inhibition of Sirt1 ubiquitination promotes Foxo3a nuclear translocation and attenuates oxidative stress injury in the kidneys of DKD mice [8].

Various glomerular and tubular lesions are closely associated with dysfunctional autophagy [39]. The Sirt1/AMP-activated protein kinase (AMPK)/mammalian target of rapamycin (mTOR) pathway reduces urinary protein levels by regulating autophagy, reducing renal inflammation, immune complex deposition and excretion, and improving renal function in systemic lupus erythematosus nephritis [40]. In addition, Sirt1 is involved in activating PTEN-induced kinase 1 (PINK1)/Parkin-associated mitochondrial autophagy and is an effective therapeutic strategy for preventing renal fibrosis [41]. H2AX phosphorylation is a key signal in the DNA damage response. It has been shown that Sirt1 directly mediates the phosphorylation of H2AX through deacetylation [42]. In addition, Sirt1 interacts with the PP4 phosphatase complex to indirectly regulate the phosphorylation of γH2AX and RPA2, ensuring comprehensive control of DNA damage [43]. Sterile alpha motif and HD domain-containing protein 1, upon deacetylation by Sirt1, binds to single-stranded DNA at the DSB, thus promoting DNA end resection and homologous recombination, and participating in the maintenance of genome stability [44] .

#### Sirt6

Sirt6 is a nuclear protein associated with DNA repair in single-strand breaks (SSBs) and DSBs. Sirt6 knockout mice exhibit chromatin abnormalities and shortened lifespans, suggesting a defects in DNA repair [45]. DNA repair efficiency decreases with age, and Sirt6 overexpression rescues senescent cells from DSB repair efficiency and improves homologous and non-homologous recombinant DSB repair pathways [46, 47]. More importantly, it has been proposed that Sirt6 is independent of known sensors and signalling pathways and is directly translocated to the site of DNA damage, accompanied by ataxia-telangiectasia mutated (ATM) kinase, recruitment of homologous recombination and non-homologous end-joining pathway proteins, and phosphorylation of H2AX, with concomitant activation of downstream pathways associated with DSB repair [48]. For example, Sirt6 coordinates with the chromatin remodeller CHD4 to promote chromatin relaxation during DNA damage, which in turn accurately regulates the process of homologous recombination and promotes the repair of DNA [49]. Sirt6 also acts synergistically with Sirt1, which deacetylates Sirt6 on residue K33, whereas the deacetylated Sirt6 is then anchored to γH2AX, which allows it to be retained in the local chromatin and remodel the chromatin [50]. Sirt6 exhibits three catalytic enzymatic activities: deacetylation, deacylation, and mono-ADP-nucleotidylation. Histone 3 lysine 9 (H3K9) and H3K56 are common histone substrates deacetylated by Sirt6 [51]. Sirt6 plays a critical role in telomere maintenance by deacetylating histone H3K9, thereby preventing telomeric DNA damage and cellular senescence [52]. Deacetylation of histone H3K56 regulates β-catenin-related genes, represses transcription of fibre-related genes, and regulates renal interstitial fibrosis [53]. Sirt6 also deacetylates non-histone proteins in the nucleus and cytoplasm, including members of the FOXO family, p53, Smad, and NAMPT [54]. Sirt6 regulates renal interstitial fibrosis by deacetylating runt-related transcription factor 2 (Runx2), promotes Runx2 translocation out of the nucleus, mediates activation of the ubiquitin-protease system, causes degradation of Runx2, and inhibits vascular calcification in chronic kidney diseases (CKD) [55]. In addition, Sirt6 physically associates with poly(ADP-ribose) polymerase 1 (PARP1) and mono-ADP-ribosylates PARP1 at lysine residue 521, thereby stimulating the poly-ADP-ribosylase activity of PARP1 and exhibiting ADP-ribosyltransferase activity [56], suggesting that Sirt6 is a multifunctional epigenetic enzyme. Additionally, Sirt6 is a target for acetylation of Sirt1, and the two act synergistically to maintain homeostasis in the organisms [57].

Sirt6 is upregulated during calorie restriction and is involved in the expression of genes involved in oxidative stress, inflammation, autophagy, and energy metabolism by regulating related targets [58]. In the glomeruli of patients with hypertensive nephropathy, an increase in DNA DSBs is accompanied by a decrease in Sirt6 expression. In contrast, overexpression of Sirt6, which increases the levels of nuclear factor-erythroid 2-related factor 2 (Nrf2), and haeme oxygenase-1 (HO-1), inhibits Ang II-induced ROS generation and DSBs in DNA and plays an essential role in alleviating Ang II stimulation-induced oxidative DNA damage [12]. Renal interstitial fibrosis is a common pathophysiological condition in chronic kidney disease. Overexpression of Sirt6 delays the progression of renal interstitial fibrosis in CKD by targeting homeodomain-interacting protein kinase 2, as evidenced by collagen deposition and reduced expression of collagen I and α-smooth muscle actin [59]. In DKD mice, Sirt6 expression is reduced, and AMPK is dephosphorylated with abnormal mitochondrial function, whereas, Sirt6 overexpression increases AMPK phosphorylation levels, suggesting that Sirt6 inhibits mitochondrial dysfunction in DKD by regulating AMPK [60]. In addition, Sirt6 overexpression also ameliorated the Ang II-induced changes in the balance between mitochondrial fusion and fission [61]. Progressive EMT in the kidneys of db/db mice is associated with Sirt6 downregulation, and reduced Sirt6 levels lead to progressive renal injury, such as tubular injury. Further studies have revealed that Sirt6 binds directly to Smad3 and, through deacetylation, inhibits its nuclear accumulation and transcriptional activity in cells and protects against renal injury in DKD [62]. In AKI, autophagy is inhibited and overexpression of Sirt6, which mediates autophagy activation, results in an increased expression of light chain 3 II and an increased lysosome/autophagosome ratio, as well as decreased p62 expression, indicating a protective effect against acute kidney injury [63]. Knockdown of Sirt6 exacerbates cisplatin-induced kidney injury, and further studies have revealed that Sirt6 binds to the promoter of extracellular signal-regulated kinase (ERK)1/ERK2 and deacetylates histone H3K9, thus inhibiting ERK1/2 expression, regulating the inflammatory response in kidney injury and providing a new therapeutic target for kidney injury under stress [64]. Sirt6 also binds to saturated fatty acids, especially palmitic acid, promoting their nuclear export, inducing acyl-CoA synthetase long-chain 5 deacetylation, and promoting fatty acid oxidation (FAO), suggesting that Sirt6 is not restricted to the nucleus to play a metabolic regulatory role and provides a reference for its study in kidney diseases [65].

#### Sirt7

Sirt7 is a nuclear-localised deacetylase that plays essential roles in inflammation, apoptosis, metabolic homeostasis, DNA damage repair, ribosome biogenesis, mitochondrial biogenesis, and glucose homeostasis [66]. Sirt7 interacts with and deacetylates HMGB1, redistributes HMGB1 to the nucleus, and activates its DNA damage repair function. Nucleophosmin (NPM), as a target of Sirt7, can be deacetylated by Sirt7, the deacetylated NPM is transferred from the nucleolus to the nucleoplasm, binds to ubiquitin ligase, and prevents ubiquitination and degradation of p53, which arrests the cell cycle and maintains the process of DNA damage repair [67]. After DNA damage, ATM activation involves autophosphorylation, and it has been proposed that deacetylation of ATM is a prerequisite for its dephosphorylation, wherwas, Sirt7 can deacetylate ATM, inhibit ATM from sustained phosphorylation and activation, and contribute to DNA damage repair [68]. In contrast, Sirt7 deficiency inhibits NF-κB phosphorylation, reduces the nuclear translocation of p53, and reduces tubular injury and renal inflammation [69]. Sirt7 directly reduces NF-κB expression, attenuates cisplatin-induced acute kidney injury, and alleviates renal tubular epithelial cell apoptosis [70]. Systemic Sirt7 knockout mice with lower renal K-Cl cotransporter (KCC)4 expression under ammonium chloride stimulation exhibited increased metabolic acidosis, and further studies have revealed that Sirt7 interacts with KCC4 to stabilise and regulate KCC4 activity through deacetylation and delays the exacerbation of renal metabolic acidosis [71]. Overexpression of Sirt7, which is accompanied by downregulation of Sirt7 levels in hypertensive kidney injury, promotes Krüppel-like factor 15/Nrf2 signalling and effectively alleviates Ang II-induced renal iron death, EMT, interstitial fibrosis, and abnormal renal function in hypertensive mice, suggesting that targeting Sirt7 is a promising strategy for the treatment of hypertensive kidney injury [72]. In addition, Sirt7-deficient mice are protected against AKI, with reduced nuclear translocation and phosphorylation of p65 and reduced inflammatory infiltration of renal cells, as evidenced by reduced proteinuria and markers of renal tubular injury [73].

### Sirtuin in the cytoplasm

#### Sirt2

Sirt2 is mainly localised in the cytoplasm but also in the mitochondria and nucleus. For example, it shuttles into the nucleus during mitosis. It is localised in the nucleus as an alternatively spliced heterodimer. In normal fibroblasts treated with nuclear export inhibitors, Sirt2 was found to be rapidly enriched in the nucleus, suggesting that nucleoplasmic shuttling may contribute to the nuclear enrichment of Sirt2 [74]. In addition, supplementation with β-NMN restored the nuclear entry of Sirt2. It rejuvenated senescent oligodendrocyte progenitors by promoting their differentiation into mature oligodendrocytes, suggesting that the nuclear entry of Sirt2 contributes to the alleviation of senescence [75].Septin4 is a pro-apoptotic protein and an important marker of organ injury, and its function is regulated by post-translational modifications. High acetylation levels at the K174 site of Septin4 exacerbated Ang II-induced oxidative stress-induced hypertensive kidney injury. In contrast, overexpressed Sirt2 interacted with the GTPase structural domain of Septin4 and caused Septin4-K174 deacetylation, which attenuated Ang II-induced hypertensive kidney injury [76]. Sirt2 regulates the acetylation state of p53 at lysine 382, contributing to the stabilisation of p53 in the nucleus, enhancing transcription, and regulating the DNA damage response [77]. Heterodimers, such as breast cancer type I susceptibility protein (BRCA1) and BRCA1-associated RING domain protein I (BARD1), are involved in homologous recombination and promote genomic integrity. It is proposed that Sirt2 binds to the BRCA1-BARD1 complex and deacetylates the conserved lysine in the BRCA1-BARD1 complex to promote BRCA1-BARD1 heterodimerization, which promotes its localization to DNA damage sites for effective homologous recombination [78]. Sirt2 is involved in the regulation of proinflammatory responses. Overexpression of Sirt2 exacerbates cisplatin-induced cellular inflammation, apoptosis, and renal injury and increases phosphorylation of p38 and c-Jun N-terminal kinase (JNK) in the kidney [79]. In contrast, Sirt2 deficiency ameliorated the lipopolysaccharide-induced infiltration of neutrophils and macrophages, and decreased renal function [80]. Further mechanistic studies revealed that knockdown of Sirt2 inhibited the phosphorylation of p38 mitogen-activated protein kinase (MAPK) and JNK. In addition, Sirt2 regulates the binding of p65 to CXCL2 and CCL2 promoters, suggesting that modulation of Sirt2 may be an important therapeutic target for inflammatory kidney injury [80]. During renal ischemia/reperfusion, activated Sirt2 binds to and deacetylates FOXO3a, promotes FOXO3a nuclear translocation, activates caspase-8 and caspase-3, and triggers apoptosis. In contrast, inhibition of Sirt2 reversed these phenomena [81]. The activity of Sirt2 contributes to the activation and proliferation of renal fibroblasts, while blocking Sirt2 activation attenuates the development of renal fibrosis and may have therapeutic potential for the treatment of CKD [82].

### Sirtuins in the mitochondria

Mitochondria are essential cellular organelles that coordinate various metabolic processes. Mitochondrial dysfunction, including altered mitochondrial biogenesis, the imbalance between fusion and division processes, oxidative stress, cytochrome c, mitochondrial DNA release, defective mitochondrial autophagy, and energy metabolism, is crucial for the pathogenesis of various renal diseases [83]. Nuclear sirtuins mainly regulate chromatin, whereas mitochondrial sirtuins mainly regulate mitochondrial proteins.

#### Sirt3

Sirt3 directly deacetylates and activates superoxide dismutase 2 (SOD2), promoting the transcription of SOD2 and peroxisomes [84]. Sirt3 also induces FOXO3a and peroxisome proliferator-activated receptor-gamma coactivator 1-alpha (PGC-1ɑ) upregulation, restoring MnSOD activity and levels [85]. It also mediates Foxo3a deacetylation and nuclear localisation, which in turn leads to the activation of Foxo3a-dependent peroxidase expression; reduces Ang II-induced renal fibrosis, endothelial-to-mesenchymal transition (EndoMT), and oxidative stress; and maintains renal endothelial homeostasis [86].

Oxidative stress is an important factors in calcium oxalate-induced kidney stone formation. Sirt3 reduces crystal deposition in the kidneys of stone model mice by regulating the Nrf2/HO-1 signalling pathway [87]. Sirt3 inhibits renal calcium oxalate crystal formation by promoting macrophage M2 polarisation via the deacetylation of FOXO1 [88]. Acetylation is an essential posttranslational modification of mitochondrial metabolism components. In the early stages of renal fibrosis, decreased Sirt3 expression is accompanied by increased mitochondrial acetylation, and Sirt3 knockout mice are prone to mitochondrial protein hyperacetylation, and severe renal fibrosis. Deacetylation of mitochondrial proteins by Sirt3 is closely associated with the remission of renal fibrosis [15]. For example, SIRT3-mediated deacetylation of OPA1 alleviates mitochondrial dysfunction in AKI mice [89]. FAO dysfunction is a crucial factor in the development of renal fibrosis. AKI mice exhibit significant FAO and lipid deposition, accompanied by high ROS production. Furthermore, deletion of Sirt3 exacerbated FAO dysfunction and kidney injury in AKI mice. Additional mechanistic studies revealed that Sirt3 may regulate FAO, repair, and delay renal injury by activating AMPK [90].

Sirt3 ameliorates pathological renal injuries such as inflammatory cell infiltration, glomerulosclerosis, and interstitial inflammation in IgAN mice by mediating autophagy to inhibit the activation of the NOD-like receptor pyrin domain containing 3 (NLRP3) inflammasome [91]. Sirt3 attenuates sepsis-induced AKI, renal tubular apoptosis, and inflammatory cytokine accumulation in the kidney, by regulating the AMPK/mTOR pathway to induce autophagy [92]. Furthermore, SIRT3 induces mitochondrial autophagy by regulating the dynamin-related protein 1 (DRP1) pathway to protect the kidney from ischemia/reperfusion injury [93]. It indirectly eliminates ROS by mediating mitochondrial autophagy and exerts antioxidant effects [94].

#### Sirt4

Sirt4 regulates the posttranslational modifications of various proteins by deacetylation, aliphatic amidase, and ADP-ribosyl/nucleotidyltransferase, thereby regulating various biological functions [95]. Glutamine metabolism plays a crucial role in cell growth, and glutamate dehydrogenase (GDH) is a critical enzyme that promotes the metabolism of glutamate and glutamine to produce adenosine triphosphate (ATP). Sirt4 promotes adenosine diphosphate (ADP) ribosylation and downregulates GDH activity, inhibiting the conversion of glutamate to α-ketoglutarate during the tricarboxylic acid cycle [96]. In addition, Sirt4 deficiency leads to decreased expression and function of the glutamate transporter [97], which may be more important than Sirt4 deacetylation. Sirt4 plays a key role in mitochondrial function and the pathogenesis of metabolic diseases, including DKD. In DKD, the mRNA and protein levels of Sirt4 are significantly decreased in glucose-mimicking podocytes in a concentration-dependent manner, and Sirt4 deficiency activates NF-κB signalling and the NLRP3 inflammasome, exacerbating renal injury [98]. In contrast, overexpression of Sirt4 decreased the expression of apoptosis-related proteins, such as Bax and phosphorylated p38, and upregulated Bcl-2 expression. It also significantly downregulates inflammatory factors, such as necrosis factor alpha (TNF-ɑ), interleukin 1 (IL)-1β, and IL-6 [99]. Increased FOXQ1 and downregulation of Sirt4 have been reported in the *db/db* mice, and overexpression of FOXQ1 further downregulated Sirt4 expression and exacerbated mitochondrial damage. In contrast, knockdown of the *FOXQ1* gene induced Sirt4 expression and partially restored mitochondrial function [100].

#### Sirt5

Sirt5 exhibits a strong affinity for negatively charged acyl groups, such as glutaric, succinic, and malonic acids; catalyses mainly lysine acylation, but also desuccinylates and deglutarylates; and has weak deacetylase activity [101]. Two key molecules regulate Sirt5 activity, and overexpression of PGC-1α elevates cellular Sirt5 levels, while activation of AMPK downregulates Sirt5 levels [102]. Elevated levels of Sirt5 in caloric restriction [103] are associated with longevity. Mice deficient in Sirt5 exhibit defective energy metabolism and reduced ATP production [104]. Mitochondrial sirtuins play key roles in mitochondria metabolism by regulating amino acid degradation, cellular respiration, ROS levels, FAO, and glycolysis [105]. Ribose-5-phosphate is required for nucleotide synthesis, and it has been found that knockdown of Sirt5 affects ribose-5-phosphate production, leading to sustained and irreparable DNA damage [106]. p53 is involved in the maintenance of genomic stabilisation. In response to DNA damage, Sirt5 mediates desuccinylation of p53 at lysine 120, thereby inhibiting p53 function [107]. Upregulation of Sirt5 expression attenuates mitochondrial dysfunction by enhancing AMPK phosphorylation, as evidenced by alleviation of mitochondrial structural damage, restoration of ATP content, and delayed AKI progression [108]. Sirt5 regulates FAO homeostasis in the mitochondria and peroxisomes in RTECs and protects against AKI injury [109]. Reduced malonylation in the renal cortex of *db/db* mice is associated with increased Sirt5 expression. Further metabolomic analysis revealed that the reduced alanine-esterified proteins were mainly enriched in non-mitochondrial metabolic pathways such as glycolysis and peroxisomal FAO. Furthermore, it has been experimentally confirmed that Sirt5 overexpression is accompanied by an increase in aerobic glycolysis, leading to altered nutrient partitioning and utilisation in DKD [110].

## Sirtuins in Kidney Disease

**Fig. 3:**
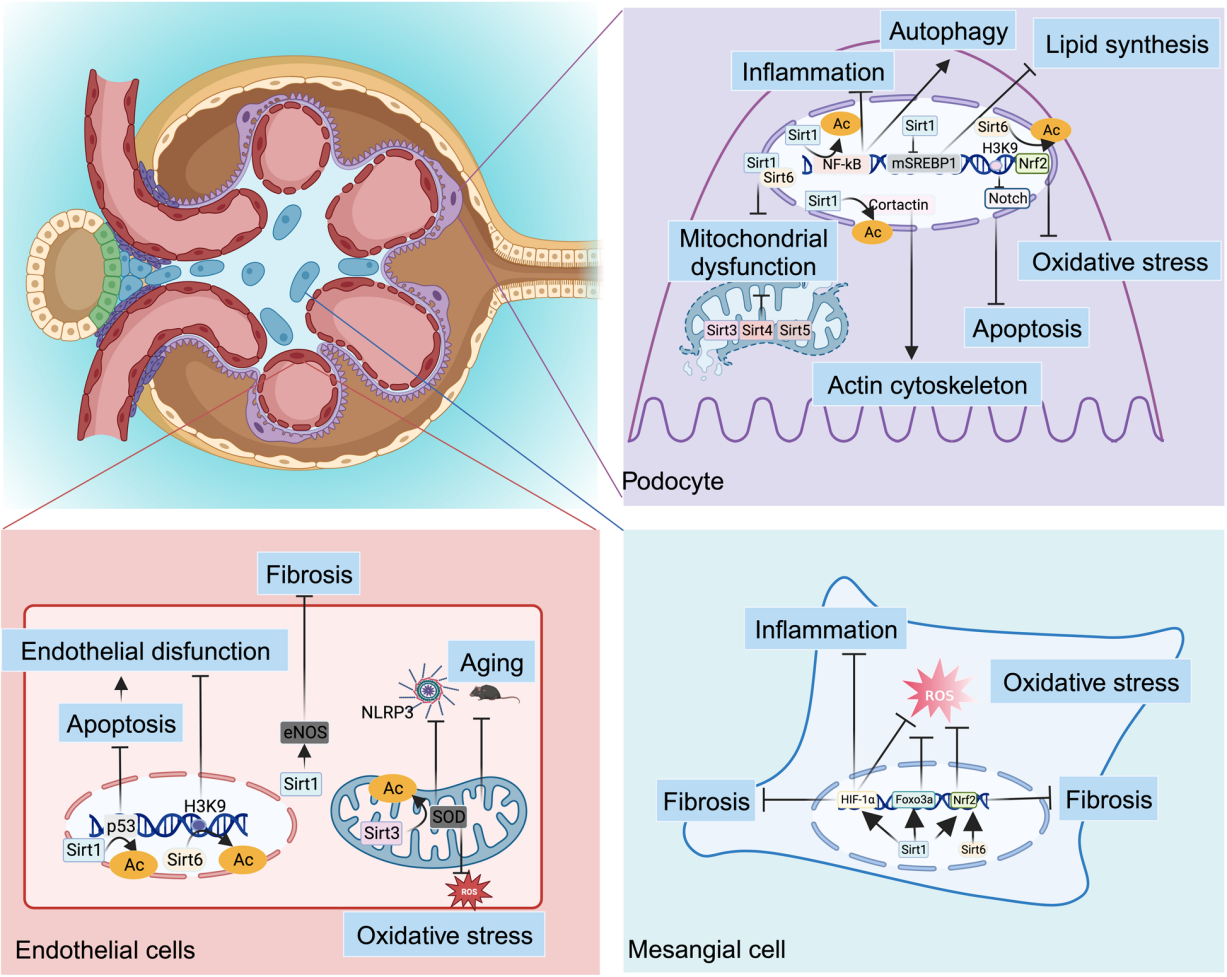
Molecular role of sirtuins in podocytes, endothelial cells, and mesangial cells. NF-κB, nuclear factor kappa B; SREBP1, sterol regulatory element-binding protein 1; H3K9, histones3 lysine9; Nrf2, nuclear factor-erythroid 2-related factor 2; eNOS, endothelial nitric oxide synthase; NLRP3, NOD-like Receptor Pyrin Domain Containing 3; SOD, superoxide dismutase; ROS, reactive oxygen species; HIF-1ɑ, hypoxia-inducible factor-1; FOXO, forkhead box O. (Created with BioRender.com)

**Fig. 4:**
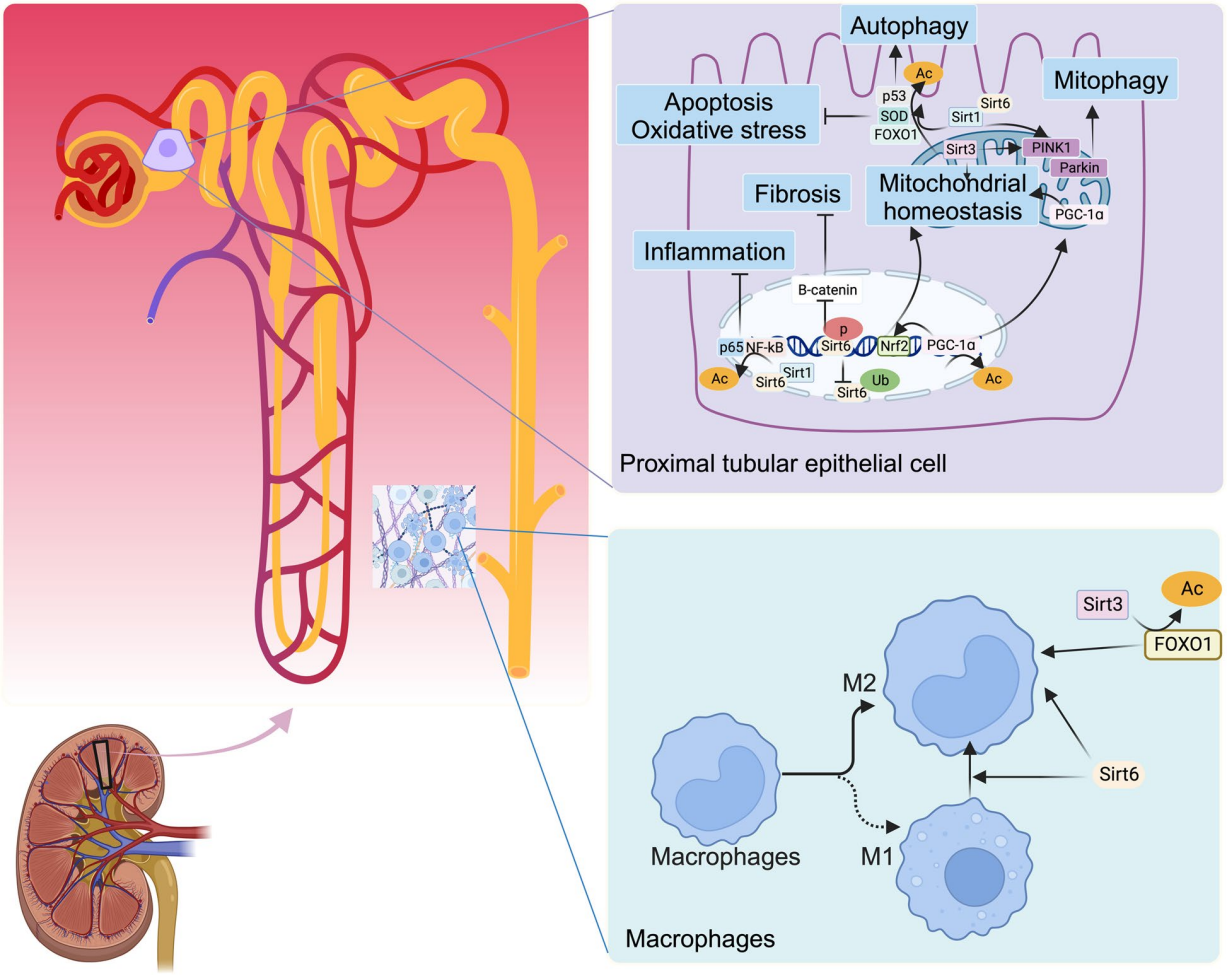
Molecular role of Sirtuins in proximal tubular epithelial cell, macrophages. SOD, superoxide dismutase; PINK1, PTEN-induced kinase 1; PGC-1ɑ, peroxisome proliferator-activated receptor-gamma coactivator 1-alpha; NF-κB, nuclear factor kappa B; Nrf2, nuclear factor-erythroid 2-related factor 2. (Created with BioRender.com)

CKD is one of the most prominent causes of death worldwide in the 21st century. The prevalence of CKD is also increasing owing increased risk factors, such as obesity and diabetes. Approximately 843.6 million people worldwide were affected by CKD in 2017. Despite the decrease in mortality of patients with end-stage renal disease, the Global Burden of Disease Organization study showed that CKD is the leading cause of death worldwide [111]. Renal diseases manifest as disorders of renal morphology structure and function, such as multiple stimuli affecting podocytes, endothelial cells, mesangial cells, and RTECs, thereby glomerulosclerosis, tubular fibrosis, proteinuria formation, and decreased renal function [112]. It is particularly important to pay attention to the regulation of renal cells [113]. The main risk factors of kidney disease include age [114], smoking, obesity [115], hypertension [116], diabetes [117], cardiovascular disease [118], hyperuricemia [119], and environmental factors [120]. Notably, sirtuins modulate most of these risk factors [23], and slow the progression of renal nephropathy by regulating metabolic homeostasis, autophagy, apoptosis, mitochondrial biogenesis, and oxidative stress; improving serum creatinine and blood urea nitrogen levels and reducing proteinuria [121–124]. Following is an overview of sirtuins in specific renal cells (Figs. 3 and 4) (Table 1).

**Table 1:** Sirtuins in renal component cells

Cell types	Sirtuins	Mechanisms
Podocytes	Sirt1, Sirt3, Sirt4, Sirt6, Sirt7	Inflammation, autophagy, lipid metabolism, mitochondrial dysfunction, actin cytoskeleton, apoptosis, oxidative stress, DNA damage, insulin resistance
Endothelial cells	Sirt1, Sirt3, Sirt6	Endothelial disfunction, inflammation, apoptosis, oxidative stress, fibrosis, aging, metabolic reprogramming
Mesangial cells	Sirt1, Sirt6	Fibrosis, inflammation, oxidative stress
Renal tubular epithelial cells	Sirt1, Sirt2, Sirt3, Sirt4, Sirt5, Sirt6, Sirt7	Inflammation, fibrosis, apoptosis, oxidative stress, autophagy, mitochondrial dysfunction, mitophagy, G2/M phase arrest
Macrophages	Sirt1, Sirt3, Sirt6	Inflammation, macrophage infiltration and activation

### Podocytes

The epithelial cells of the visceral glomerular layer, i.e., podocytes, are terminally differentiated cells that emit secondary protrusions (foot process) that interlock and occlude each other to form a “zipper-like” septum structure, which together with endothelial cells and glomerular basement membrane forms the glomerular filtration barrier and maintains normal filtration function. Under the influence of external factors such as mechanical stress, immune mediators, oxidative stress, and abnormal accumulation of metabolites, podocytes are damaged, resulting in structural changes in the septum protein complex, dysfunction of the actin skeleton, and damage to the top negative charge barrier, leading to increased podocyte activity, fusion of the foot process and increased apoptosis [125]. When damaged podocytes are shed, parts of the basement membrane are exposed, glomerular filtration barrier integrity is disrupted, and a high degree of proteinuria develops [126]. Various CKD cases with proteinuria as the primary manifestation, including microscopic lesion nephropathy, focal segmental glomerulosclerosis, membranous nephropathy, immunoglobulin A (IgA) nephropathy, and DKD, are closely associated with podocyte injury [127, 128].

Sirtuins exert pleiotropic protective effects on podocytes, including inflammation, autophagy, lipid metabolism, mitochondrial dysfunction, apoptosis, and oxidative stress. Mice with podocyte-specific Sirt1 knockdown increase their inflammation-related markers and exacerbate NLRP3 inflammatory vesicle activation, leading to increased glomerulosclerosis and proteinuria [129, 130]. The above phenomena can be reversed by Sirt1 overexpression [131], consistent with Sirt4 studies, where Sirt4 activation inhibits NF-κB signalling and NLRP3 inflammatory vesicles, increases podocyte nephrin expression and decreases podocyte pyroptosis [98]. Similarly, Sirt6-deficient mice exhibit more severe podocyte hypertrophy, loss of peduncles, and reduced septin cleavage, exacerbating the progression of proteinuria [132]. Activation of Sirt1 reduces the acetylation of NF-κB p65, increases beclin1 expression, promotes autophagy, and reduces EMT [133]. Sirt6 deacetylates histone H3K9 to inhibit the transcription of Notch1 and Notch4 and the Notch pathway to enhance autophagy [14]. Sirtuins are key transcription factors that regulate lipid metabolism. Biological analysis of clinical samples suggests that Sirt6 is involved in Ang II-induced glomerular cholesterol dysregulation and that Sirt6 deficiency in podocytes exacerbates Ang II-induced renal injury and attenuates urinary protein. Sirt6 affects cholesterol efflux in podocytes by regulating the expression of ATP-binding cassette transporter G1 [134]. The reduction of Sirt1 increases sterol regulatory element binding protein 1 (SREBP1) acetylation, which induces lipid synthesis and phosphorylates SREBP1, thus eliminating the inhibition of lipid synthesis [135]. In addition, Sirt1 mediates PGC-1α inhibition of acetyl-CoA carboxylase 2, attenuating HG-induced insulin resistance and lipotoxicity-mediated damage to podocytes [136].

Increasing evidence has shown that mitochondrial dysfunction is a crucial driver of HG-induced podocyte injury. Sirt1 expression has been reported to be reduced in HG-treated podocytes, and phosphorylation levels are significantly upregulated at the S47 locus, accompanied by downregulation of synaptopodin and nephrin. ROS levels and cytochrome c release exacerbate mitochondrial dysfunction [137]. Sirt6 knockdown exacerbated HG-induced reduction of mitochondrial numbers, increased mitochondrial superoxide production, and decreased mitochondrial membrane potential, which exacerbated mitochondrial division through DRP1 phosphorylation, whereas Sirt6 overexpression increased AMPK phosphorylation, attenuated HG-induced apoptosis of podocytes and oxidative stress, and improved Ang II-induced changes in the balance between mitochondrial fusion and division [60, 61]. In addition, Sirt1 protects podocytes by deacetylating cortactin, thereby maintaining actin cytoskeleton integrity [32]. Sirt3 acts as a mitochondrial sirtuin, and Sirt3-deficient mice exhibit earlier and more severe proteinuria with podocyte and mitochondrial dysfunction after a high-fat diet [138]. Silencing Sirt7 promoted HG-induced podocyte apoptosis, whereas Sirt7 overexpression attenuated it [139]. Overexpression of Sirt4 also inhibited apoptosis, downregulated the expression of Bax and phosphorylated p38, upregulated the expression of Bcl-2, increased mitochondrial membrane potential, and reduced ROS production, in addition to also significantly attenuating the inflammatory response, as evidenced by decreased levels of TNF-ɑ, IL-1β, and IL-6 [99]. DNA DSBs are closely associated with the development of renal disease. In the glomeruli of patients with hypertensive nephropathy, an increase in DNA DSBs is accompanied by a decrease in Sirt6 expression. Similar results have been observed in rat kidneys infused with Ang II and in cultured podocytes stimulated with Ang II. In contrast, Sirt6 overexpression inhibited Ang II-induced ROS generation and DNA DSBs, thus protecting against Ang II-induced podocyte apoptosis [12]. Compared to other sirtuins, Sirt5 has been less studied in kidney podocytes.

### Endothelial cells

Endothelial cells in the kidney, podocytes, and the basement membrane form the glomerular filtration membrane barrier. Endothelial cells are also a part of the renal vasculature. Defects in the endothelium of the kidney lead to changes in its structure and function, thereby disrupting the glomerular filtration barrier and contributing to the formation of proteinuria. Sirt1 depletion in vascular endothelial cells mediates endothelial dysfunction and premature aging in renal disease. Mice with endothelial Sirt1-specific knockouts exhibit impaired endothelium-dependent vasodilation and angiogenesis, and low levels of fibrosis can spontaneously develop at a young age [140]. Furthermore, endothelial Sirt1 dysfunction leads to the activation of endothelial notch1 signalling, resulting in peritubular capillary sparing and fibrosis after kidney injury. In contrast, overexpression of Sirt1, which inhibits notch1 signalling, antagonizes fibrosis [141]. Sirt1 in vascular smooth muscle cells reprograms endothelial cells to inhibit angiogenesis after ischemia [142], which maintains the differentiated phenotype of vascular smooth muscle cells and protects them from stress-induced vascular remodelling.

Recently, EndoMT has been recognised as a critical factor in promoting fibrosis in chronic kidney disease. In hypertensive kidney injury, Sirt3 expression is significantly reduced, accompanied by an increase in EndoMT induction, ROS, renal fibrosis, and renal inflammatory cell infiltration, as well as decreased telomerase expression [143], which is also consistent with the findings of Sirt7 [144]. Although endothelial cells overexpressing Sirt3 reduced Ang II-induced renal fibrosis and EndoMT, further mechanistic studies revealed that this was achieved through the SIRT3-Foxo3a-peroxidase pathway, thereby maintaining endothelial homeostasis [86]. Sirt1 promotes p53 deacetylation, reduces p53 deacetylation levels, upregulates Bax and Bcl-2 levels, and reduces apoptosis [145], in addition to increasing the level of phosphorylated endothelial nitric oxide synthase [38] and inhibiting EndoMT development. Sirt6 deacetylates histone H3K9, inhibits NK3 homeobox 2 transcription, induces the expression of GATA-binding protein 5 (GATA5), which is a novel regulator of blood pressure, and reduces endothelial cell senescence. It promotes autophagy and prevents endothelial damage [146]. Endothelial cell Sirt3 deficiency also stimulates transforming growth factor beta (TGF-β)/Smad3-dependent mesenchymal transition in RTECs, thereby contributing to metabolic reprogramming and fibrosis [147].

### Mesangial cells

Glomerular mesangial cells (GMCs) are stromal cells that are important for internal environmental stability and injury response. Increasing evidence suggests that MCs, such as stromal fibroblasts, pericytes, and vascular smooth muscle cells, determine tissue architecture and regulate developmental processes and cell fates. Furthermore, by crosstalk with adjacent cells and indirectly through stromal remodelling, stromal cells can regulate various processes, such as immune, inflammatory, regenerative, and maladaptive fibrotic responses. MCs support capillaries within the glomerulus and extend into the extraglomerular region called extraglomerular mesangial cells [148]. Most studies to date have proposed that sirtuins are beneficial to the kidney. Treatment of GMCs with advanced glycation end-products resulted in decreased protein expression and activity of Sirt1, accompanied by increased levels of fibronectin (FN) and TGF-β1 in a dose- and time-dependent manner, and inhibition of Sirt1 activity further induced the production of FN and TGF-β1. In contrast, overexpression of Sirt1 significantly enhanced the activity of the kelch-like ECH-associated protein 1 (Keap1)/Nrf2/antioxidant response element (ARE) pathway, including decreasing the expression of Keap1; promoting the ability and transcriptional activity of Nrf2 to bind to the ARE; increasing the protein level of HO-1, a target gene of Nrf2; and ultimately inhibiting the overproduction of ROS and alleviating the accumulation of FN and TGF-β1 in GMCs of advanced glycation end-products (AGEs)-treated GMCs [149, 150]. The effects of Sirt6 were similar to those of Sirt1, and the excessive upregulation of Sirt6 effectively inhibited proliferation, migration, fibrosis, and the inflammatory response in high glucose-induced rat mesangial cells [151]. Sirt1 in MCs directly induces Foxo3a to exert antioxidant effects and attenuate oxidative stress damage in GMCs [8]. Overexpression of Sirt1, which inhibits HIF-1ɑ expression, suppresses inflammation and fibrosis in rat GMCs cultured with HG [152]. However, it has also been proposed that upregulation of Sirt1 expression in MCs promotes cyclooxygenase-2 expression, enhances prostaglandin E2 biosynthesis, and promotes glomerular inflammation [153].

### Renal tubular epithelial cells

Under physiological conditions, Small protein molecules are reabsorbed by the proximal renal tubular epithelium. In progressive kidney disease, the degree of proteinuria is positively correlated with the degree of tubular damage, and structural changes and dysfunction of the tubules due to various factors play essential roles in the decline in renal function caused by proteinuria, which is an important driver of the progression of chronic kidney disease. Calorie restriction contributes to elevated levels of Sirt1 expression in RTECs [154]. Renal fibrosis is an essential pathological change associated with progressive kidney disease and EMT is a crucial features of renal fibrosis. The deletion of Sirt6 in proximal RTECs exacerbates UUO-induced tubular injury and ECM deposition, and further studies have revealed that proximal tubular Sirt6 may play an essential role in UUO-induced tubular interstitial inflammation and fibrosis by regulating Sirt6-dependent β-catenin acetylation and ECM protein promoter transcription [155]. Sirt1 plays a key role in UUO-induced tubular interstitial inflammation and fibrosis by deacetylating FoxO1 to inhibit the ROS pathway and by deacetylating Smad4 to inhibit the TGFβ/Smad pathway [156], in addition to reducing HIF-1ɑ activity by deacetylating HIF-1ɑ and decreasing the expression of ECM components, such as FN, collagen type I and collagen type IV, ultimately reducing renal EMT and diabetic tubulointerstitial fibrosis [157].

Sirt6 depletion exacerbates hypoxia-induced renal tubular injury and G2/M phase arrest. Sirt6 overexpression has been reported to attenuate hypoxia-induced injury and G2/M phase arrest in RTECs [158]. Autophagy is a cellular self-renewal process that requires lysosomal degradation and is used to maintains cellular energy homeostasis. p53 deacetylation is promoted by Sirt1, which enhances autophagy in RTECs and attenuates sepsis-induced AKI [159]. In addition, Sirt1 is involved in PINK1/Parkin-related activation of mitochondrial autophagy and inhibits apoptosis and scorching of RTECs, thereby reducing sepsis-induced AKI [160]. Sirt3 induces autophagy by regulating the AMPK/mTOR pathway, thereby protecting the renal tubular epithelium against caecal ligation and puncture-induced damage to the renal tubular epithelium [92]. RTECs require high levels of energy and are dependent on the mitochondria for their energy supply. Sirt3, 4, and 5 are sirtuins localised in the mitochondria and are closely associated with renal tubular epithelial injury and repair. In DKD, Sirt4 expression is decreased with mitochondrial dysfunction [100]. Sirt5 depletion impairs ATP production, decreases mitochondrial membrane potential, and drives mitochondrial division in RTECs [161]. Sirt5 regulates the balance between mitochondrial and peroxisomal FAO in proximal RTECs to protect against AKI [109]. Sirt1, through deacetylation, activates PGC-1α, induces Nrf1 production, and participates in mitochondrial biogenesis [162]. The Sirt1/p53 axis also decreases mitochondrial swelling and mitochondrial cristae disorganisation, increases mitochondrial membrane potential, and elevates ATP content [163]. Sirt3 induces mitochondrial autophagy, fusion, and division through the regulation of DRP1 pathway homeostasis and mitochondrial dynamics to protect the kidney from ischaemia-reperfusion injury [93, 164, 165].

In contrast to other sirtuin members, Sirt2 regulates proinflammatory immune responses. When Sirt2 is activated during renal ischemia/reperfusion, it can bind to and deacetylate FOXO3a, thereby enhancing FOXO3a nuclear translocation, accompanied by caspase-8 and caspase-3 activation, thus promoting apoptosis of RTECs [81]. Inhibition of Sirt2 expression promotes the expression of mitogen-activated protein kinase phosphatase-1 and downregulates JNK and p38 phosphorylation, thereby alleviating renal tubular epithelial cell apoptosis, pyroptosis, and inflammation [79]. Similarly, in mice with Sirt7-specific knockout in RTECs, ischaemia-reperfusion resulted in reduced proteinuria, tubular injury markers, and inflammatory infiltration [73].

### Macrophages

Macrophages are present in the glomeruli and interstitium at all stages of renal disease. In DKD mice, the accumulation and activation of macrophages triggered glomerular and tubular damage, induced renal inflammation, and increase the expression of fibrotic factors [166]. Furthermore, exosomes from RTECs contribute to macrophage infiltration and activation, thus providing new insights into renal tubular interstitial macrophages [167]. Interestingly, the number of tubulointerstitial macrophages predicts renal dysfunction compared to glomerular macrophages [168]. Renal macrophages play an essential role in the pathogenesis of kidney disease and are potential therapeutic targets for kidney injury and fibrosis [169]. White adipose tissue plays an essential role in the development of renal metabolic disorders, and increasing the activity of Sirt1 by activators alleviates the free fatty acid (FFA)-induced inflammatory response in macrophages and inflammation in white adipose tissue [170]. Sirt3 inhibits the formation of renal calcium oxalate crystals by promoting M2 polarization via the deacetylation of FOXO1 [88]. Similarly, overexpression of Sirt6 promotes M2 macrophage conversion and alleviates renal injury in patients with DKD. In vitro experiments with macrophages and podocytes found that glucose promoted macrophage M1 transformation and podocyte apoptosis in a dose-dependent manner and attenuated Sirt6 expression. After successful transfection of macrophages with the Sirt6-overexpression plasmid, macrophages were transformed into the M2 phenotype, and Sirt6 was overexpressed in podocytes. Furthermore, in the Transwell™ co-culture system, Sirt6 overexpression in macrophages, but not Sirt6 overexpression in podocytes, protected podocytes from HG-induced injury. However, apoptosis of podocytes overexpressing Sirt6 (induced by transfection with Sirt6-overexpression plasmid) remained elevated when co-cultured with macrophages in HG medium. Sirt6 has been reported to protect podocytes from injury in a simulated DKD microenvironment by activation of M2 macrophages [171].

## Sirtuin regulators

Given that sirtuins are involved in a variety of cell-mediated biological processes in the kidney and can serve as targets for the prevention and treatment of age-related diseases, including kidney disease, the following is an overview of selected sirtuin modulators that are of greatest relevance to kidney disease (Tabl 2).

**Table 2:** Sirtuins as modulators in kidney diseases

Classification	Name	Structure	Target	Cell	Disease		Effect	Pathway	References
Natural sirtuin agonists	Resveratrol		Sirt1	Podocyte	DKD	Mitochondrial oxidative stress		Sirt1/PGC-1α	[172]
			Sirt1	/	Cadmium-induced nephrotoxicity	Mitophagy		Sirt1/PINK1/Parkin	[173]
	Curcumin		Sirt1	RTECs	Aristolochic acid nephropathy	Oxidative stress		Sirt1/Nrf2/HO-1	[174]
	Silybin		Sirt3	RTECs	Cisplatin-induced AKI	Mitochondrial dysfunction, apoptosis		/	[175]
	Honokiol		Sirt3	RTECs	Cisplatin-induced AKI	Mitochondrial fission		Sirt3/AMPK	[176]
	Quercetin		Sirt1	RTECs	Senescence and renal fibrosis	Mitophagy		Sirt1/PINK1/Parkin	[41]
	Isoliquiritigenin		Sirt1	NRK-52E cells	STZ-induced DKD	Inflammation, oxidative stress		Sirt1/MAPKs, Sirt1/Nrf2	[177]
			Sirt1	/	STZ-induced DKD	Inflammation		Sirt1/NF-κB; Sirt1/NLRP3	[178]
Synthetic sirtuin agonists	SRT1720		Sirt1	Podocytes	DKD	Autophagy		Sirt1/NF-κB p65	[133]
	SRT3025		Sirt1	NRK-49F cells	Senescence and renal fibrosis	Fibrogenesis		Sirt1/TGF-β	[179]
	MDL-800		Sirt6	RTECs	UUO	Inflammation, Fibrosis		Sirt6/β-Catenin;TGF-β1/Smad	[155]
	SRT2183		Sirt1	Renal medullary interstitial cells	UUO	Oxidative stress, apoptosis, fibrosis		Sirt1/COX2	[180]
NAD^+^ promoter	NMN		Sirt1	Podocytes	Focal glomerulosclerosis	Histone methylation		NMN/NAD	[181]
					DKD	Histone methylation		Sirt1/Claudin-1	[182]
	NR		Sirt3	Podocytes	CKD	Mitochondrial Dysfunction, Oxidative stress		Sirt3/PGC-1α	[183]
Sirtuins inhibitors	AGK2		Sirt2	RTECs	AKI	Apoptosis		Sirt2/FOXO3a	[81]
			Sirt2	NRK-49F cells	UUO	Fibrosis		Sirt2/EGFR/PDGFRβ	[82]

*Abbreviations*: *NMN* nicotinamide mononucleotide, *NR* nicotinamide riboside, *RTECs* renal tubular epithelial cells, *DKD* diabetic kidney disease, *AKI* acute kidney injury, *STZ* streptozotocin, *UUO* unilateral ureteral obstruction, *CKD* chronic kidney diseases, *PGC-1ɑ* peroxisome proliferator-activated receptor-gamma coactivator 1-alpha, *PINK1* PTEN-induced kinase 1, *Nrf2* nuclear factor-erythroid 2-related factor 2, *HO-1* heme oxygenase-1, *AMPK* AMP-activated protein kinase, *MAPK* mitogen-activated protein kinase, *NLRP3* NOD-like Receptor Pyrin Domain Containing 3, *NF-κB* nuclear factor kappa B, *TGF-β* transforming growth factor beta, *COX2* cyclooxygenase-2, *NRK-49F* cultured renal interstitial fibroblasts, *EGFR* epidermal growth factor receptor, *PDGFRβ* platelet-derived growth factor receptor-β

### Natural sirtuin agonists

Natural products have a rich history of use as treatments for various human diseases. Most sirtuin activators are natural polyphenolic products, and resveratrol was the first natural Sirt1 activator to be reported [184]. Resveratrol attenuates proteinuria and reduces malondialdehyde levels in diabetic mice, in addition to increasing renal cortical Mn-SOD activity, inhibiting apoptosis of glomerular podocytes and RTECs, improving pathological manifestations, and restoring Sirt1 and PGC-1α expression in the renal tissues of DKD mice. In HG-exposed podocytes, resveratrol inhibited the production of excess ROS and apoptosis. In addition, resveratrol directly reduces mitochondrial ROS production, improves the activity of respiratory chain complexes I and III, increases mitochondrial membrane potential, and inhibites the release of cytochrome C from the mitochondria to the cytoplasm [172]. Other polyphenols and natural compounds, such as curcumin, silybin, honokiol, and quercetin, have also been shown to regulate sirtuins. Curcumin, a polyphenol isolated from turmeric, regulates oxidative stress and mitochondrial damage and delays the onset and progression of aristolochic acid nephropathy by activating the SIRT1/Nrf2/HO-1 signalling pathway [174]. Silymarin, a pharmacological activator of Sirt3, can protect against cisplatin-induced apoptosis of RTECs and AKI by improving mitochondrial function [175]. As a small-molecule polyphenol, treatment with honokiol restored Sirt3 expression, improved AMPK activity in RTECs exposed to cisplatin, preserved DRP1 phosphorylation at Ser637, and prevented its translocation into mitochondria, thereby preventing mitochondrial fragmentation and subsequent cell injury and death [176]. Quercetin has been reported to reduce RTECs senescence and alleviate renal fibrosis by activating Sirt1/PINK1/Parkin-mediated mitochondrial phagocytosis [41]. Isoliquiritigenin, a natural flavonoid dependent on Sirt1, protects against DKD injury and inhibits inflammation and oxidative stress. Molecular docking has demonstrated that isoliquiritigenin binds directly to Sirt1 and regulates the MAPK and Nrf-2 signalling pathways to neutralise inflammatory responses and oxidative stress and reverse the deterioration of renal function and renal fibrosis [177]. Isoliquiritigenin also inhibits inflammation by activating Sirt-1 and regulating the activities of NF-κB and NLRP3, thereby attenuating collagen deposition in DKD and preserving renal structure and function [178].

### Synthetic sirtuin agonists

Given the critical role of sirtuin activation in age-related diseases including kidney diseases, many sirtuin-related compounds with high affinities have been synthesised. Examples include SRT1720, SRT3025, and MDL-800. SRT1720 activates Sirt1 and has been reported to reduce p65 acetylation, enhance autophagy in HG-induced podocyte EMT, reverse renal fibrosis, and improve renal function [133]. SRT3025 also activates Sirt1 and has been reported to reverse the increase in collagen production due to TGF-β1 stimulation, reduce glomerulosclerosis and tubulointerstitial fibrosis, and attenuate the decrease in the glomerular filtration rate and proteinuria [179]. Additionally, as an activator of Sirt1, SRT2183 increased the tolerance of renal medullary interstitial cells to oxidative stress and reduced renal apoptosis and fibrosis in a mouse model of UUO kidney injury through a Sirt1-mediated increase in cyclooxygenase-2 (COX2) expression in renal medullary interstitial cells [180]. The Sirt6 activator MDL-800 has been reported to attenuate UUO-induced tubulointerstitial inflammation and fibrosis. In vitro experiments have shown that MDL-800 reduced TGF-β1-induced myofibroblast activation and ECM production by modulating Sirt6-dependent β-catenin acetylation and the TGF-β1/Smad signalling pathway [155].

### NAD^+^ promoter

Sirtuins play an essential role by activating the conversion of NAD^+^ to NAM, which then becomes NMN through the action of the transferase iNAMPT, which in turn can be converted to NAD^+^, forming a cycle in which NAD^+^ plays an important role [5]. Two methods exist to enhance the level of NAD^+^; direct restoration of NAD^+^ levels by NAD^+^ precursor supplementation, and overexpression of two enzymes related to NAD^+^ synthesis, NAMPT and NMNAT, to enhance the rate of NAD^+^ synthesis. The NAD^+^ precursor supplements include NMN and nicotinamide riboside (NR). NMN attenuated the rate of NAD^+^ synthesis in adriamycin-treated mice with increased urinary albumin excretion, attenuated glomerulosclerosis, reduced Sirt1 expression, and elevated claudin-1 expression in mouse kidneys. In addition, the NAD^+^ concentration in the kidney increased [181]. Short-term NMN supplementation increases renal NAD^+^ concentration, enhances Sirt1 function, and alleviates the onset of DKD by downregulating Claudin-1 expression through an epigenetic mechanism [182]. Furthermore, oral administration of NMN supplementation to healthy volunteers for 12 weeks caused no abnormalities in physiological and laboratory tests, and no significant adverse effects were observed. The NAD^+^ levels in whole blood increased significantly after NMN administration [185]. NR supplementation proved beneficial in AKI with ischaemia‒reperfusion injury, as evidenced by a slowing increase of serum urea nitrogen and creatinine levels and tubular damage [186]. Restoration of Sirt3 activity, restoration of reduced glomerular numbers, improvement of glomerular podocyte density, and sparse density of renal capillaries by administration of NR supplementation to mothers on a low-protein diet provides a therapeutic option to potentially limit the long-term sequelae of a reduced kidney number in adulthood [183]. Intracellular NAMPT is a critical enzyme in the NAD^+^ remediation synthesis pathway. However, it enables NAD^+^ self-cycling, so activation of NAMPT may combat aging-related diseases. For example, NATs, which are more efficient and have better bioavailability than NMN and NR, are regulated at rate-limiting steps, allowing more flexibility to meet the needs of cells in different physiological states [187].

### Sirtuins inhibitors

In addition to sirtuin activators, several sirtuin-inhibiting compounds have been developed to treat various renal diseases. Studies have confirmed the involvement of Sirt2 in renal fibrosis and inflammation, and inhibitors of Sirt2, such as AGK2, and AK-1, have been developed. Administration of the Sirt2 inhibitor AGK2 prior to renal ischaemia-reperfusion significantly reduced the number of apoptotic renal tubular cells and attenuated ultrastructural damage [81]. The activity of Sirt2 may contribute to the activation and proliferation of renal fibroblasts, and the Sirt2 inhibitor AGK2 inhibited renal fibroblast activation and, to a lesser extent, cell proliferation in a dose- and time-dependent manner, as evidenced by reduced expression of α-smooth muscle actin, collagen I, and fibronectin [82]. AK-1 also inhibits Sirt2 to increase Nrf2 activity and downregulates JNK signalling to reduce oxidative stress [188].

## Conclusion

Sirtuins are essential guardians of life and health by maintaining genomic stability and protecting cells and organisms from various stresses. Furthermore, most sirtuin deficiencies lead to cell structure and functional disorders that can promote the development of various kidney diseases. As summarized above, sirtuins are present at abnormal levels in various renal diseases, such as AKI, DKD, and CKD, as well as in various cells of renal diseases, such as podocytes, mesangial cells, endothelial cells, and RTECs. Moreover, regulation of the expression or activity of sirtuins has been shown to delay disease progression in both cellular and animal models.

Over the past decade, significant efforts have been made to develop effective and safe sirtuin modulators. Some sirtuins agonists have gradually moved from preclinical to clinical studies, offering new possibilities for small-molecule drugs targeting sirtuins. Although sirtuin activators and NAD^+^ promoters have yielded promising results in terms of improving various indicators in preclinical studies, such as markers of pathological damage in podocytes and RTECs, there is no substantial evidence to date that these approaches can improve the progression of human kidney disease or prevent the occurrence of such events. More importantly, the pharmacokinetics and therapeutic potential of these sirtuin modulators in renal disease are unclear, the pathways through which sirtuins act remain to be elucidated, and the safety of these drugs is pending the evaluation of more extended treatment regimens.

However, the targeting of sirtuins to regulate renal disease has been mainly been studied in specific cell types. Nevertheless, the kidney, as a specific tissue, is a highly cooperative system of various cell types, and the various mechanisms do not work in isolation but rather interact and collaborate. In recent years, an increasing number of studies have started to focus on the crosstalk mechanisms between different cells in the kidney. For example, Sirt1 in RTECs alleviates diabetic proteinuria by epigenetically suppressing claudin-1 overexpression in podocytes, and Sirt1 in RTECs protects diabetic patients from proteinuria by maintaining the NMN concentration around the glomerulus, thereby affecting podocyte function [189]. Many common signalling pathways in renal cells interact through multiple small molecules, exosomes, and cytokines to produce acute biological effects during the formation and progression of various pathological processes. However, targeting sirtuins to regulate the communication between renal cells requires further study.

## Data Availability

No data were used for the research described in the article.

## Abbreviations

NAD^+^: Nicotine adenine dinucleotide
Sir2: Silent information regulator 2
NAM: Nicotinamide
NMN: Nicotinamide mononucleotide
iNAMPT: Intracellular nicotinamide phosphoribosyltransferase
NMNAT: Nicotinamide mononucleotide adenylyltransferase
DKD: Diabetic kidney disease
AKI: Acute kidney injury
FOXO: Forkhead box O
OPA1: Optic atrophy 1
DSBs: Double-strand breaks
Ang II: angiotensin II
ROS: Reactive oxygen species
RTECs: Renal tubular epithelial cells
HG: High glucose
STZ: Streptozotocin
YY1: Yin yang 1
EMT: Epithelial-mesenchymal transition
HMGB1: High-mobility group box 1
ECM: Extracellular matrix
HIF-1α: Hypoxia-inducible factor-1
NF-κB: Nuclear factor kappa B
STAT: Signal transducer and activator of transcription
UUO: Unilateral ureteral obstruction
eNOS: Endothelial nitric oxide synthase
AMPK: AMP-activated protein kinase
mTOR: Mammalian target of rapamycin
PINK1: PTEN-induced kinase 1
SSBs: Single-strand breaks
ATM: Ataxia-telangiectasia mutated
H3K9: Histones3 lysine9
Runx2: Runt-related transcription factor 2
CKD: Chronic kidney diseases
PARP1: Poly (ADP-ribose) polymerase 1
Nrf2: Nuclear factor-erythroid 2-related factor 2
HO-1: Heme oxygenase-1
ERK: Extracellular signal-regulated kinase
FAO: Fatty acid oxidation
NPM: Nucleophosmin
KCC: K-Cl cotransporter
BRCA1: Breast cancer type I susceptibility protein
BARD1: BRCA1-associated RING domain protein I
JNK: c-Jun N-terminal kinase
MAPK: Mitogen-activated protein kinase
SOD: Superoxide dismutase
PGC-1ɑ: Peroxisome proliferator-activated receptor-gamma coactivator 1-alpha
EndoMT: Endothelial-to-mesenchymal transition
NLRP3: NOD-like receptor family pyrin domain containing 3
DRP1: Dynamin-related protein 1
GDH: Glutamate dehydrogenase
ATP: Adenosine triphosphate
ADP: Adenosine diphosphate
TNF-ɑ: Tumor necrosis factor-alpha
IL: Interleukin
IgA: Immunoglobulin A
SREBP1: sterol regulatory element binding protein 1
GATA5: GATA-binding protein 5
TGF-β: Transforming growth factor beta
GMCs: Glomerular mesangial cells
FN: Fibronectin
ARE: Antioxidant response element
Keap1: Kelch-like ECH-associated protein 1
AGEs: Advanced glycation end-products
FFA: Free fatty acid
NR: Nicotinamide riboside
